# Single nucleotide polymorphisms associated with risk for contralateral breast cancer in the Women's Environment, Cancer, and Radiation Epidemiology (WECARE) Study

**DOI:** 10.1186/bcr3057

**Published:** 2011-11-17

**Authors:** Sharon N Teraoka, Jonine L Bernstein, Anne S Reiner, Robert W Haile, Leslie Bernstein, Charles F Lynch, Kathleen E Malone, Marilyn Stovall, Marinela Capanu, Xiaolin Liang, Susan A Smith, Josyf Mychaleckyj, Xuanlin Hou, Lene Mellemkjaer, John D Boice, Ashley Siniard, David Duggan, Duncan C Thomas

**Affiliations:** 1 Center for Public Health Genomics, University of Virginia, P.O. Box 800717, Charlottesville, VA, 22908-0717, USA; 2 Department of Biochemistry and Molecular Genetics, University of Virginia, P.O. Box 800733, Charlottesville, VA, USA; 3 Department of Epidemiology and Biostatistics, Memorial Sloan-Kettering Cancer Center, 307 East 63rd Street, 3rd Floor, New York, NY, 10065, USA; 4 Department of Preventive Medicine, Norris Comprehensive Cancer Center, University of Southern California, 1441 Eastlake Avenue, Los Angeles, CA, 90033, USA; 5 Division of Cancer Etiology, Department of Population Sciences, Beckman Research Institute, City of Hope, 1500 East Duarte Road, Duarte, CA, 91010, USA; 6 Department of Epidemiology, University of Iowa, E220 General Hospital, Iowa City, IA, 52242, USA; 7 Division of Public Health Sciences, Fred Hutchinson Cancer Research Center, 1100 Fairview Avenue N, Seattle, WA, 98109, USA; 8 Department of Radiation Physics, The University of Texas M.D. Anderson Cancer Center, 1515 Holcombe Blvd. unit 544, Houston, TX, 77030, USA; 9 Department of Public Health Sciences, P.O. Box 800717, University of Virginia, Charlottesville, VA, 22908-0717, USA; 10 Institute of Cancer Epidemiology, Danish Cancer Society, Strandboulevarden 49, DK-2100, Copenhagen, Denmark; 11 International Epidemiology Institute, 1455 Research Boulevard, Suite 550, Rockville, MD, 20850-6115, USA; 12 Division of Epidemiology, Department of Medicine, Vanderbilt Epidemiology Center, Vanderbilt-Ingram Cancer Center, Vanderbilt University School of Medicine, 691 Preston Building, Nashville, TN, 37232-6838, USA; 13 Translational Genomic Research Institute (TGen), 445 N. Fifth Street, Phoenix, AZ, 85004, USA

## Abstract

**Introduction:**

Genome-wide association studies, focusing primarily on unilateral breast cancer, have identified single nucleotide polymorphisms (SNPs) in a number of genomic regions that have alleles associated with a significantly increased risk of breast cancer. In the current study we evaluate the contributions of these previously identified regions to the risk of developing contralateral breast cancer. The most strongly disease-associated SNPs from prior studies were tested for association with contralateral breast cancer. A subset of these SNPs, selected upon their main effects on contralateral breast cancer risk was further evaluated for interaction with treatment modalities and estrogen receptor (ER) status.

**Methods:**

We genotyped 21 SNPs in 708 women with contralateral breast cancer and 1394 women with unilateral breast cancer who serve as the cases and controls in the Women's Environment, Cancer and Radiation Epidemiology (WECARE) Study. Records of treatment and ER status were available for most of WECARE Study participants. Associations of SNP genotypes and risk for contralateral breast cancer were calculated with multivariable adjusted conditional logistic regression methods.

**Results:**

Multiple SNPs in the *FGFR2 *locus were significantly associated with contralateral breast cancer, including rs1219648 (per allele rate ratio (RR) = 1.25, 95%CI = 1.08-1.45). Statistically significant associations with contralateral breast cancer were also observed at rs7313833, near the *PTHLH *gene (per allele RR = 1.26, 95%CI = 1.08-1.47), rs13387042 (2q35) (per allele RR = 1.19, 95%CI = 1.02-1.37), rs13281615 (8q24) (per allele RR = 1.21, 95%CI = 1.04-1.40), and rs11235127 near *TMEM135 *(per allele RR = 1.26, 95%CI = 1.04-1.53). The A allele of rs13387042 (2q35) was significantly associated with contralateral breast cancer in ER negative first tumors while the A allele of rs11235127 (near *TMEM135) *was significantly associated with contralateral breast cancer in ER positive first tumors. Although some SNP genotypes appeared to modify contralateral breast cancer risk with respect to tamoxifen treatment or particular radiation doses, trend tests for such effects were not significant.

**Conclusions:**

Our results indicate that some common risk variants associated with primary breast cancer also increase risk for contralateral breast cancer, and that these risks vary with the ER status of the first tumor.

## Introduction

Patients with breast cancer are two to five times more likely to develop a second primary cancer in the contralateral breast than are unaffected women to develop an initial breast cancer [1-4]. Established risk factors for asynchronous second primary contralateral breast cancer (CBC) include those suggesting a genetic basis, such as early age at diagnosis or family history [1], as well as exogenous factors such as the treatment for the first breast cancer [5,6]. Prior genome-wide association studies (GWASs) have identified regions of the genome containing single-nucleotide polymorphisms (SNPs) with alleles that are associated with an increased risk for breast cancer [7-9]. Many of these SNPs were also reported to be associated with estrogen receptor (ER)-positive breast cancer [9-11]. For the most part, the specific causative variants in these regions and their mechanisms of action remain to be elucidated. In the present study, we evaluated the contributions of these identified loci to risk for CBC.

Our study builds on an existing, population-based, epidemiologic study of CBC: the Women's Environment, Cancer, and Radiation Epidemiology (WECARE) Study. The WECARE study is a nested case-control study in which women with CBC (*n *= 708) serve as cases and women with unilateral breast cancer (UBC) (*n *= 1,394) serve as controls [12]. This study population is well characterized for treatment-related exposures, including radiation therapy (RT) (including dose to the contralateral breast), chemotherapy, and hormonal therapy, enabling examination of the interaction of genetic risk factors and cancer therapy. Previous reports from the WECARE Study have shown that RT for a first cancer was associated with an increased risk of CBC [6] but that chemotherapy and tamoxifen treatment were associated with a lower risk of CBC [5]. These treatment effects were further modulated by genetic background. For example, missense variants in the *ATM *gene were associated with an increased risk of CBC in women who were treated with radiation for their first primary breast tumors [13].

In the present study, we genotyped 21 SNPs representing 18 genomic regions in the WECARE Study population. These SNPs were selected on the basis of their having attained either genome-wide significant evidence or suggestive evidence of association with breast cancer in a prior GWAS that included at least one replication in a separate population. Furthermore, we assessed the interaction of these SNPs with radiation dose to the contralateral breast, chemotherapy, tamoxifen therapy, and ER status.

## Materials and methods

### Study population

The WECARE Study is a multicenter, population-based, nested case-control study of 708 cases (women with asynchronous bilateral breast cancer) and 1,394 controls (women with UBC) recruited through five population-based cancer registries: one registry covering all of Denmark and, in the US, one registry covering Iowa, two registries covering three counties in Southern California, and one registry covering three counties in Washington State. The controls were individually matched 2:1 to cases on date and age at diagnosis of the first primary breast tumors, race, and registry region and were counter-matched on registry-reported RT. Each case-control triplet consists of two women who received RT and one who did not, maximizing informativeness for radiation exposure [12]. For 12 cases with only one matched control, matched pairs consisted of one exposed and one unexposed woman [12]. The WECARE Study protocol was approved by the institutional review board at each site and the ethics committee system in Denmark, and informed consent was obtained from all participants.

### Treatments and phenotypes

Medical records were retrieved to obtain detailed information on the treatment of the first breast cancer (chemotherapy, hormonal therapy, and radiotherapy) during the at-risk period (time between first and second primary diagnoses) and other tumor characteristics, including ER status [5,6].

### Radiation dosimetry

Estimated absorbed radiation doses to various specific contralateral breast locations were reconstructed for each treatment regimen by using tissue-equivalent phantoms and modeling procedures as described previously [6,12]. Dosage information was available for 604 to 607 cases and 1,184 to 1,195 controls, depending on the number of genotyped individuals per SNP.

### Single-nucleotide polymorphism selection

Twenty-one SNPs were selected from among those most associated with breast cancer in three prior GWASs [7-9]. All of these prior studies included at least one replication in a separate population. Nine of the SNPs were associated with breast cancer in prior studies at genome-wide significance levels (*P *< 10^-7^). These were rs2981582, rs12443621, rs8051542, rs889312, rs3817198, and rs13281615 from Easton and colleagues [7]; rs1219648 from Hunter and colleagues [8]; rs13387042 from Stacey and colleagues [9]; and rs3803662 reported in both Stacey and colleagues [9] and Easton and colleagues [7]. The remaining 12 SNPs were described as showing evidence of association (global *P *= 0.001 in three populations) in Easton and colleagues [7] and were listed in the supplemental materials of those authors.

### Genotyping

Multiplex SNP genotyping was carried out with the Illumina Golden Gate™ assay on the Sentrix Array Matrix scanned with the Bead Array Reader (Illumina Inc., San Diego, CA, USA). To avoid any bias in the calling of genotypes, all DNA samples were coded and laboratory personnel were blinded to case-control status. A blinded 24% re-sampling was carried out for quality control purposes and these samples were interspersed throughout the plates. Negative controls lacking DNA were included on each plate. Results from a subset of SNPs previously genotyped by an individual SNP method, the MGB Eclipse™ Probe System (Epoch Biosciences, ELITech Group, Paris, France), were concordant with the multiplex genotyping results. Samples with more than 10% missing genotypes and SNPs with more than 5% missing genotypes were excluded from analysis. Although Hardy-Weinberg equilibrium may not strictly apply given that all participants in the study were affected with breast cancer, SNPs deviating from Hardy-Weinberg equilibrium with a *P *value of less than 0.001 were also excluded.

### Haplotyping

Common SNPs (minor allele frequency of greater than 0.1) in the *FGFR2 *intron 2 region near SNP rs2981582 were selected from the HapMap Project [14]. The Tagger program within Haploview [15] was used to select SNPs informative for the region with an *r^2 ^*of greater than 0.8. The program PLINK [16] was used to estimate phase. These tagging SNPs were genotyped with the MGB Eclipse™ Probe System. In the haplotype analysis, the referent group for each haplotype consists of those without that haplotype.

### Statistical analysis

The WECARE Study is based on a standard nested case-control design with the additional feature of counter-matching on radiotherapy status. All analyses were conducted with multivariable adjusted conditional logistic regression methods, incorporating an 'offset term' or weight to adjust appropriately for the counter-matched sampling [12]. For the SNP main effects analysis, *P *value for trend was from the log-additive model. The test for trend in Table 1 assessed the hypothesis that CBC risk increases with radiation dose and risk allele 'dose'. Trend tests had 1 degree of freedom. All analyses were performed with SAS (Statistical Analysis System) (SAS Institute Inc., Cary, NC, USA).

**Table 1:** Risk of contralateral breast cancer associated with radiation dose by single-nucleotide polymorphism genotype

		Homozygous; reference allele			Heterozygous			Homozygous; risk allele			***P *value**^**e**^
SNP^a^/gene region	**RT dose**^**b**^	Cases^c ^(CBC)	Controls (UBC)	RR^d ^(95% CI)	Cases^c ^(CBC)	Controls (UBC)	RR^d ^(95% CI)	Cases^c ^(CBC)	Controls (UBC)	RR^d ^(95% CI)	
rs2981582/*FGFR2*	0	93	75	1.0	139	119	1.0	66	39	1.0	
	0 < 1.0	40	172	0.9 (0.6-1.5)	88	253	1.3 (0.9-1.9)	40	108	1.0 (0.6-1.8)	0.39
	1.0+	41	131	1.2 (0.8-2.0)	74	212	1.4 (1.0-2.1)	25	85	0.8 (0.4-1.4)	
rs1219648/*FGFR2*	0	80	74	1.0	147	117	1.0	71	42	1.0	
	0 < 1.0	40	165	1.1 (0.7-1.8)	85	256	1.2 (0.8-1.7)	44	112	1.1 (0.7-1.9)	0.37
	1.0+	34	124	1.3 (0.8-2.2)	79	216	1.4 (1.0-2.0)	27	89	0.8 (0.5-1.5)	
rs3803662/*TOX3*	0	116	115	1.0	141	97	1.0	40	21	1.0	
	0 < 1.0	85	238	1.6 (1.1-2.3)	66	242	0.9 (0.6-1.3)	17	52	0.9 (0.4-2.0)	0.07
	1.0+	63	199	1.5 (1.0-2.3)	57	175	1.1 (0.7-1.6)	19	54	0.8 (0.4-1.8)	
rs13281615/8q24	0	89	67	1.0	141	128	1.0	70	39	1.0	
	0 < 1.0	45	159	1.0 (0.6-1.6)	80	269	1.2 (0.9-1.7)	42	103	1.1 (0.6-1.9)	0.99
	1.0+	41	132	1.2 (0.7-2.0)	71	226	1.3 (0.9-1.8)	28	71	1.2 (0.6-2.1)	
rs13387042/2q35	0	66	55	1.0	147	114	1.0	86	62	1.0	
	0 < 1.0	31	118	1.1 (0.6-1.8)	75	259	1.1 (0.8-1.5)	61	155	1.3 (0.8-2.1)	0.45
	1.0+	34	107	1.1 (0.6-1.9)	59	202	1.1 (0.8-1.7)	46	120	1.4 (0.9-2.2)	
rs11235127	0	205	162	1.0	81	66	1.0	13	5	1.0	
/*TMEM135*	0 < 1.0	109	374	1.1 (0.8-1.5)	50	144	1.3 (0.8-2.1)	9	15	0.7 (0.2-3.1)	0.78
	1.0+	91	306	1.2 (0.9-1.6)	39	108	1.3 (0.8-2.3)	9	13	1.1 (0.3-4.3)	
rs7313833/*PTHLH*	0	124	103	1.0	134	97	1.0	41	31	1.0	
	0 < 1.0	57	232	1.0 (0.6-1.4)	83	257	1.1 (0.8-1.6)	27	40	2.3 (1.1-4.9)	0.42
	1.0+	56	194	1.1 (0.8-1.7)	62	185	1.2 (0.8-1.8)	21	45	1.7 (0.8-3.6)	
rs7696175/4p14	0	114	72	1.0	127	110	1.0	58	51	1.0	
	0 < 1.0	65	185	1.0 (0.7-1.6)	77	242	1.4 (1.0-2.0)	26	103	0.9 (0.5-1.7)	0.97
	1.0+	49	141	1.1 (0.7-1.7)	69	208	1.5 (1.0-2.1)	21	79	1.0 (0.5-1.8)	
rs2107425/H19	0	132	96	1.0	137	110	1.0	29	27	1.0	
	0 < 1.0	86	265	1.1 (0.8-1.6)	64	211	1.1 (0.7-1.6)	16	56	1.4 (0.6-3.1)	0.14
	1.0+	60	217	0.9 (0.6-1.4)	66	167	1.6 (1.1-2.3)	14	43	1.5 (0.6-3.5)	

^a^Single-nucleotide polymorphisms (SNPs) that had a main effect on contralateral breast cancer (CBC) risk with a *P *value of less than 0.1 were selected for radiation therapy (RT) dose interaction analysis (rs2107425 did not meet this criterion but is included because of a report [36] that occupational radiation dose affects rs2107425-associated breast cancer risk). ^b^Radiation dose to the contralateral breast (mea*n *= 1.1 Gy) in the quadrant where the second primary tumor occurred. ^c^Total number of case subjects and matched control subjects for whom there were location-specific dose estimates ranged from 604 to 607 cases and 1,184 to 1,195 controls, depending on the number of genotyped subjects per SNP. ^d^Rate ratio (RR) adjusted for age at diagnosis of first primary tumor and the weighting factor that accounts for the counter-matched design. ^e^One degree of freedom trend test for modification of radiation dose effect by SNP genotype. CI, confidence interval; UBC, unilateral breast cancer.

## Results

The risk of CBC associated with each of the 21 SNPs from 18 genomic regions selected from prior GWASs of UBC [7-9] is shown in Table 2. We observed statistically significant associations with CBC at four of nine SNPs previously reported to be associated with breast cancer at genome-wide significance levels (*P *< 10^-7^): rs2981582 (per-allele rate ratio (RR) = 1.20, 95% confidence interval (CI) = 1.04 to 1.40) and rs1219648 (per-allele RR = 1.25, 95% CI = 1.08 to 1.45) at 10q26 in the *FGFR2 *gene, rs13281615 in the 8q24 region (per-allele RR = 1.21, 95% CI = 1.04 to 1.40), and rs13387042 in the 2q35 region (per-allele RR = 1.19, 95% CI = 1.02 to 1.37). We observed statistically significant associations with CBC at two of the remaining 12 SNPs: rs7313833, located on chromosome 12p in the vicinity of the *PTHLH *gene (per-allele RR = 1.26, 95% CI = 1.08 to 1.47), and rs11235127 on chromosome 11q14.2 near *TMEM135 *(per-allele RR = 1.26, 95% CI = 1.04 to 1.53). Excluding *BRCA1/2 *carriers (who constitute 5.4% of the WECARE Study population [17,18]) from the present analysis did not markedly alter the results for any of the SNPs tested here (data not shown).

**Table 2:** Risk of contralateral breast cancer associated with single-nucleotide polymorphisms reported in prior breast cancer genome-wide association studies

**SNP**^**a**^	Gene region	Genotype	Cases (CBC)	Controls (UBC)	Per-allele rate ratio^b ^(95% CI) trend	Heterozygous rate ratio^b ^(95% CI)	Homozygous rate ratio^b ^(95% CI)	***P *value**^**c**^
rs2981582	*FGFR2*	GG	204	449	1.20 (1.04-1.40)	1.26 (0.99-1.60)	1.45 (1.08-1.95)	0.01
		AG	351	672				
		AA	149	268				
rs1219648	*FGFR2*	AA	183	433	1.25 (1.08-1.45)	1.36 (1.07-1.74)	1.55 (1.15-2.08)	0.003
		AG	360	676				
		GG	162	281				
rs12443621	*TOX3*	GG	173	350	1.03 (0.89-1.19)	1.03 (0.80-1.32)	1.06 (0.79-1.42)	0.70
	(*TNRC9*)	AG	355	710				
		AA	177	326				
rs8051542	*TOX3*	GG	215	421	0.96 (0.82-1.11)	0.81 (0.64-1.04)	0.94 (0.70-1.27)	0.57
	(*TNRC9*)	AG	344	709				
		AA	144	261				
rs3803662	*TOX3*	CC	306	640	1.16 (0.99-1.36)	1.08 (0.87-1.35)	1.43 (1.01-2.01)	0.06
	(*TNRC9*)	CT	309	606				
		TT	88	143				
rs889312	*MAP3K1*	AA	343	658	0.99 (0.85-1.17)	0.95 (0.76-1.18)	1.02 (0.70-1.47)	0.93
		AC	296	597				
		CC	67	131				
rs3817198	*LSP1*	AA	320	650	1.08 (0.93-1.27)	1.12 (0.90-1.40)	1.12 (0.79-1.60)	0.31
		AG	309	600				
		GG	76	140				
rs2107425	*H19*	CC	325	673	0.97 (0.83-1.14)	1.01 (0.81-1.25)	0.88 (0.62-1.26)	0.74
		CT	311	569				
		TT	67	146				
rs13281615	8q24	AA	201	420	1.21 (1.04-1.40)	1.06 (0.83-1.35)	1.49 (1.11-2.01)	0.01
		AG	338	720				
		GG	166	250				
rs30099	5q11	GG	571	1,125	0.92 (0.72-1.18)	0.99 (0.76-1.30)	0.50 (0.18-1.35)	0.50
		AG	128	244				
		AA	8	15				
rs4666451	2p24	GG	286	524	0.95 (0.82-1.10)	0.86 (0.68-1.08)	0.96 (0.71-1.31)	0.49
		AG	310	669				
		AA	106	197				
rs13387042^d^	2q35	GG	152	326	1.19 (1.02-1.37)	1.12 (0.86-1.47)	1.39 (1.04-1.85)	0.02
		AG	327	669				
		AA	225	391				
rs11235127	*TMEM135*	GG	476	977	1.26 (1.04-1.53)	1.12 (0.88-1.41)	2.29 (1.32-4.00)	0.02
		AG	194	373				
		AA	35	39				
rs7313833	*PTHLH*	GG	272	616	1.26 (1.08-1.47)	1.24 (1.00-1.55)	1.65 (1.18-2.31)	0.003
		AG	329	625				
		AA	103	139				
rs16998733	4q31	GG	556	1,071	0.87 (0.69-1.10)	0.77 (0.59-1.00)	1.48 (0.65-3.37)	0.25
		AG	134	291				
		AA	11	21				
rs1318703	16p31	AA	240	485	1.40 (0.90-1.20)	1.06 (0.84-1.34)	1.11 (0.82-1.50)	0.63
		AG	327	646				
		GG	133	234				
rs4331913	5p13	GG	247	454	0.98 (0.84-1.14)	0.94 (0.75-1.19)	0.98 (0.72-1.33)	0.77
		AG	343	700				
		AA	116	230				
rs4954956	2p21	GG	367	748	1.03 (0.88-1.21)	1.03 (0.83-1.28)	1.06 (0.72-1.57)	0.71
		AG	287	532				
		AA	53	109				
rs6469633	8q23	TT	383	791	1.05 (0.88-1.25)	1.11 (0.89-1.38)	0.96 (0.62-1.50)	0.58
		TC	278	509				
		CC	43	86				
rs981782	5p12	AA	243	423	0.90 (0.78-1.05)	0.84 (0.67-1.07)	0.84 (0.62-1.12)	0.18
		AC	334	693				
		CC	127	271				
rs7696175	4p14	GG	268	457	0.88 (0.76-1.02)	0.89 (0.70-1.11)	0.77 (0.57-1.04)	0.09
		AG	319	663				
		AA	118	266				

^a^Single-nucleotide polymorphisms (SNPs) selected from among the most significantly associated with breast cancer in the genome-wide association studies by Easton and colleagues [7], Hunter and colleagues [8], and Stacey and colleagues [9] described further in the 'Single-nucleotide polymorphism selection' section of Materials and methods. ^b^Rate ratio adjusted for age at diagnosis of first primary tumor and counter-matching weight. ^c^*P *value with 1 degree of freedom for the association of per-allele SNP genotype and contralateral breast cancer (CBC) risk. ^d^For rs13387402, A is not the minor allele in this study population. CI, confidence interval; UBC, unilateral breast cancer.

To refine the observed association between CBC and SNPs in the vicinity of *FGFR2*, we genotyped five haplotype-tagging SNPs, in addition to rs2981582, in all 2,102 available WECARE Study participants and found alleles at four of the six SNPs that were individually associated with risk of CBC (Additional file 1, Table S1). Whereas the risk alleles at these SNPs occurred individually on a number of haplotypes, they occurred together on only two, of which only one was significantly associated with an increased risk of CBC (Additional file 1, Table S2).

For SNPs with a *P *value of less than 0.1 for main effects in Table 2, we carried out additional exploratory analyses considering the effect on CBC risk of treatment or tumor characteristics of the first cancer. Previous studies have shown that SNP alleles with main effects on the risk for breast cancer can sometimes be associated with specific subtypes of breast cancer [10,11,19]. In Table 3, the association of SNPs with CBC was assessed for heterogeneity with respect to the ER status of the first primary tumor. The risk variant at rs13387402 (2q35) was associated with CBC only in individuals with ER-negative first tumors (interaction *P *value = 0.0008). The risk variant at rs11235127 (near *TMEM135*) was associated with CBC only in individuals with ER-positive first tumors (interaction *P *value of not more than 0.01).

**Table 3:** Risk of contralateral breast cancer associated with single-nucleotide polymorphism genotypes according to estrogen receptor status

		**ER-positive**^**a**^			**ER-negative**^**a**^			
SNP^b^/Gene region	Genotype	Cases (CBC)	Controls (UBC)	RR^c ^(95% CI)	Cases (CBC)	Controls (UBC)	RR^c ^(95% CI)	***P *value**^**d**^
rs2981582/*FGFR2*	GG	96	232	1.0	64	124	1.0	
	AG	167	347	1.3 (0.9-1.9)	100	165	1.3 (0.8-2.0)	0.99
	AA	73	161	1.6 (1.0-2.3)	28	49	1.3 (0.7-2.3)	
rs1219648/*FGFR2*	AA	81	221	1.0	58	119	1.0	
	AG	174	354	1.4 (1.0-2.0)	102	169	1.4 (0.9-2.2)	0.74
	GG	80	164	1.7 (1.1-2.6)	33	50	1.5 (0.8-2.8)	
rs3803662/*TOX3*	CC	138	331	1.0	89	156	1.0	
	CT	150	335	1.2 (0.9-1.7)	81	137	1.1 (0.7-1.7)	0.26
	TT	47	75	1.8 (1.1-2.9)	23	43	0.9 (0.5-1.7)	
rs13281615/8q24	AA	97	222	1.0	51	100	1.0	
	AG	158	378	1.1 (0.8-1.5)	103	175	1.3 (0.8-2.1)	0.78
	GG	82	141	1.4 (0.9-2.1)	38	62	1.2 (0.6-2.2)	
rs13387042/2q35	GG	83	169	1.0	31	85	1.0	
	AG	141	347	0.8 (0.6-1.2)	105	177	1.8 (1.1-3.1)	0.0008
	AA	112	224	0.9 (0.6-1.3)	57	73	3.1 (1.7-5.7)	
rs11235127/*TMEM135*	GG	212	538	1.0	138	218	1.0	
	AG	108	180	1.5 (1.1-2.1)	46	106	0.8 (0.5-1.2)	0.01
	AA	17	22	2.5 (1.2-5.3)	9	12	1.0 (0.3-3.0)	
rs7313833/*PTHLH*	GG	116	337	1.0	92	155	1.0	
	AG	168	326	1.5 (1.1-2.0)	76	147	0.9 (0.6-1.4)	0.30
	AA	52	71	2.0 (1.2-3.3)	25	32	1.4 (0.7-2.8)	
rs7696175/4p14	GG	145	246	1.0	63	111	1.0	
	AG	141	357	0.8 (0.6-1.0)	97	169	1.0 (0.6-1.6)	0.39
	AA	51	135	0.7 (0.4-1.0)	33	55	1.0 (0.5-1.8)	

^a^Estrogen receptor (ER) status of the first primary tumor. ^b^Single-nucleotide polymorphisms (SNPs) that had a main effect on contralateral breast cancer (CBC) risk with a *P *value of less than 0.1 were selected for ER status interaction analysis. ^c^Rate ratio (RR) adjusted for age at diagnosis of first primary tumor and weighting factor that accounts for the counter-matched design. ^d^One degree of freedom interaction *P *value. CI, confidence interval; UBC, unilateral breast cancer.

There were no significant modifying effects of SNP genotype on CBC risk associated with radiation dose to the contralateral breast for those patients receiving RT for their first cancer (Table 1). Although individual genotypes for SNPs rs3803662 near *TOX3 *(*TNRC9*), rs7313833 near *PTHLH*, and rs2107425 (*H19*) appeared to be associated with increased risk for CBC in particular dose-allele combinations (for example, for rs7313833 minor allele homozygotes at 0.01 to 0.99 Gy radiation, RR = 2.3, 95% CI = 1.1 to 4.9), there were no obvious trends by either gene or radiation dose. Finally, none of the SNPs studied significantly modified the effects of tamoxifen use or chemotherapy (data not shown) on the incidence of CBC.

## Discussion

Breast cancer GWASs have tended to focus on high-risk individuals such as women from multiple-case breast cancer families or with early age at onset. The present study extends findings from these studies by examining another group of genetically predisposed women, those with CBC, and considers important treatment co-factors such as RT, chemotherapy, and hormonal exposures as well as ER status of the first tumor.

We observed nominally significant associations between CBC and SNPs in three regions previously associated with breast cancer, 10q26 (*FGFR2*), 8q24, and 2q35, all of which had been previously replicated in studies of UBC [20-28]. Rs13387042 (2q35) was also more strongly associated with bilateral than unilateral disease in a recent meta-analysis [28].

Our initial genotyping data revealed an association between an SNP in the *FGFR2 *gene (rs2981582) and CBC with an RR of a magnitude similar to that observed in the original GWAS report [7]. Further mapping of the *FGFR2 *intron 2 region with haplotype-tagging SNPs in the WECARE Study population suggested that increased risk for CBC was not restricted to rs2981582 but was associated with a single haplotype, carrying risk alleles at multiple SNPs, consistent with other reports [29,30].

Among SNPs genotyped in the WECARE Study which were as yet unconfirmed in other studies, the strongest associations with CBC risk were at rs7313833 on chromosome 12p11.22 and at rs11235127 on chromosome 11q14.2. For both of these SNPs, risk was particularly elevated in homozygotes for the minor allele. Although these two SNPs were among the 30 most significantly associated with breast cancer risk in a prior GWAS [7], the associations had not been previously replicated in other study populations [31]. The SNP rs7313833 falls within a small region that is very highly conserved among mammals but that provides no evidence of being a coding region itself. The nearest flanking genes are *PTHLH*, parathyroid hormone-like hormone, which is located 28 kb centromeric to the reference SNP, and *KLHDC5*, Kelch domain-containing protein 5, which is located 127 kb telomeric to the reference SNP. In HapMap CEU (Utah residents with Northern and Western European ancestry from the Centre d'Etude du Polymorphism Humain collection) **{ **data, rs7313833 is contained within a region of linkage disequilibrium extending in the centromeric direction to include the relatively small *PTHLH *gene. *PTHLH *has a broad range of functions, including roles in mammary gland development and tumor progression [32,33], making it a potential candidate gene. Annotation of *KLHDC5 *suggests no obvious connection to breast development or breast cancer.

On chromosome 11, rs11235127 is not located in an evolutionarily conserved region, but there are several uncharacterized, apparently spliced ESTs (expressed sequence tags) in the region. The nearest annotated gene is *TMEM135*, which is located 54 kb centromeric to the reference SNP and which encodes a transmembrane protein that is not well characterized. The nearest gene in the telomeric direction is *RAB38*, a Ras family member that is expressed exclusively in melanocytes and that is located more than 700 kb away. We considered *TMEM135 *to be a candidate gene since studies in the mouse indicate that it is transcriptionally regulated upon treatment with tamoxifen, an estrogen analog and therapeutic agent that is associated with a decreased risk of CBC in the WECARE Study population [34]. However, we observed no statistically significant modifying effect of alleles at rs11235127, the SNP located near *TMEM135*, or any other SNP studied on the effect of tamoxifen on CBC risk.

In WECARE Study participants who had received RT, the mean radiation dose to the contralateral breast during treatment was 1.1 Gy. In our previous studies of this population, we observed that women who were under 40 years of age and who received more than 1.0 Gy of absorbed dose to a specific quadrant of the contralateral breast had a significant, 2.5-fold greater risk for CBC developing in that quadrant than unexposed women [6]. The presence of specific variants in the *ATM *gene further increased the risk of CBC specifically in those women who received RT for their first cancer. Accordingly, in the present study, we assessed whether radiation dose-associated CBC risk was modified by genotype; owing to small sample numbers in individual categories, we could not further stratify by age. Overall, the tests for interaction were not statistically significant, although some specific genotype-dose combinations were. For rs3803662 near the *TOX3 *gene, homozygotes for the reference allele had a statistically significantly increased risk for CBC with radiation dose, whereas carriers of the minor allele did not show statistically significantly increased risk of CBC or dose response. Currently, the function of *TOX3 *is not well understood and its relevance to radiation response is not clear. The only evident connection between *TOX3 *and breast cancer is that the gene has been implicated in breast tumor metastasis to bone [35]. Homozygotes for the risk allele at rs7313833, near *PTHLH*, who received RT had a significantly increased risk of CBC. Interestingly, *PTHLH *has also been implicated in breast tumor metastasis to bone in a mouse model [32]. Owing to a report that genotype-associated breast cancer risk varied significantly by occupational radiation dose (mean of less than 0.05 Gy) for rs2107425 in *H19 *[36], we evaluated CBC risk by radiation dose (mean of 1.1 Gy) and rs2107425 genotype. Heterozygotes who received at least 1.0 Gy were at increased risk for CBC, but there was no statistically significant trend with dose and genotype.

Previous studies have assessed whether SNP associations with breast cancer vary with ER status: rs2981582 (in *FGFR2*), rs3803662 in (*TOX3*), rs13387042 (in 2q35), and rs13281615 (in 8q24) were more strongly associated with ER-positive disease than ER-negative disease [9,10,27]. In the WECARE Study, only rs13387042 (in 2q35) and rs11235127 (near *TMEM135*) associations with CBC risk displayed statistically significant heterogeneity with regard to ER status. In our study, 27.4% of the CBC cases had an ER-negative first primary tumor and 23.6% of the UBCs were ER-negative overall [17]. The A allele at rs13387042 was associated with increased risk of CBC in participants with ER-negative first tumors. Although an earlier study reported this allele to be associated with ER-positive disease [9], more recent meta-analyses have observed significant associations with both ER-positive and ER-negative disease [27,37]. Differences in results between the present study and previous studies could reflect specificity to CBC. No previous study has examined rs11235127 (near *TMEM135*) with regard to ER status.

While the WECARE Study was designed to examine potential interaction effects of genetic risk factors with treatment exposures and tumor characteristics, there are limitations to its application in the context of common risk factors with modest effects. The WECARE Study does not include an unaffected (non-cancer) control population; controls in the study are women with diagnosed UBC. Although women with CBC represent a high-risk breast cancer group, the power to detect main effects of common risk alleles on CBC is limited by the likely enrichment for these same alleles among the UBC patients who serve as controls in the study.

## Conclusions

Our study demonstrates that a subset of common variants with moderate effects on breast cancer risk is also associated with an increased risk for CBC. In particular, we identified nominally significant associations with CBC for six of 21 SNPs implicated in prior GWASs of UBC. Two of these SNPs were significantly associated with CBC risk, depending on ER status of the first tumor. Particular combinations of SNP genotypes with radiation dose were associated with increased risk for CBC, although there were no statistically significant interactions with increasing dose and genotype. These findings underscore the need to consider breast cancer tumor characteristics and treatment modalities when examining the risk associated with individual SNPs in order to help characterize their role in breast carcinogenesis.

## Abbreviations

CBC: contralateral breast cancer; CI: confidence interval; ER: estrogen receptor; GWAS: genome-wide association study; RR: rate ratio; RT: radiation therapy; SNP: single-nucleotide polymorphism; UBC: unilateral breast cancer; WECARE: Women's Environment: Cancer: and Radiation Epidemiology.

## Competing interests

LB is an editor of *Breast Cancer Research*. All other authors declare that they have no competing interests.

## Authors' contributions

SNT participated in study design, data acquisition (molecular genetics) and interpretation, and manuscript drafting and revision. JLB participated in study design, manuscript revision, and study supervision. ASR and MC participated in statistical analysis and interpretation of the data and in manuscript revision. RWH, LB, CFL, MS, SAS, and LM participated in data acquisition and manuscript revision. KEM participated in data acquisition and manuscript revision and provided critical manuscript revision. XL, JM, and XH participated in data analysis and manuscript revision. AS and DD participated in data acquisition (molecular genetics) and manuscript revision. JDB participated in data interpretation and critical manuscript revision. DCT participated in statistical analysis and interpretation and in critical manuscript revision. PC participated in study design, data interpretation, critical manuscript revision, and study supervision. The WECARE Study Collaborative Group participated in recruitment and data acquisition. All authors read and approved the final manuscript.

## WECARE Study Collaborative Group

Memorial Sloan-Kettering Cancer Center (New York, NY): Jonine L Bernstein (WECARE Study principal investigator), Colin Begg, Marinela Capanu, Xiaolin Liang, Jennifer Brooks, and Anne R Reiner

City of Hope (Duarte, CA) (some work performed at the University of Southern California, Los Angeles, CA): Leslie Bernstein and Laura Donnelly-Allen

Danish Cancer Society (Copenhagen, Denmark): Jørgen H Olsen, Michael Andersson, Lisbeth Bertelsen, Per Guldberg, and Lene Mellemkjær

Fred Hutchinson Cancer Research Center (Seattle, WA): Kathleen E Malone

International Epidemiology Institute (Rockville, MD) and Vanderbilt University (Nashville, TN): John D Boice Jr.

Lund University (Lund, Sweden): Åke Borg

Mount Sinai School of Medicine (New York, NY): Barry S Rosenstein and David P Atencio

National Cancer Institute (Bethesda, MD): Daniela Seminara

New York University (New York, NY): Roy E Shore

Norwegian Radium Hospital and University of Oslo (Oslo, Norway): Anne-Lise Børresen-Dale

Translational Genomics Research Institute (TGen) (Phoenix, AZ): David Duggan and Ashley Siniard

University of California at Irvine (Irvine, CA): Hoda Anton-Culver (sub-contract principal investigator) and Joan Largent

University of California at Los Angeles (Los Angeles, CA): Richard A Gatti

University of Iowa (Iowa City, IA): Charles F Lynch

University of Southern California (Los Angeles, CA): Robert W. Haile, Graham Casey, Daniel Stram, Duncan C Thomas, Jane Figueiredo, Shanyan Xue, Nianmin Zhou, and Anh T Diep

University of Southern Maine (Portland, ME): W Douglas Thompson

The University of Texas, M.D. Anderson Cancer Center (Houston, TX): Marilyn Stovall and Susan Smith

University of Virginia (Charlottesville, VA) (some work performed at Benaroya Research Institute at Virginia Mason, Seattle, WA): Patrick Concannon, Sharon N Teraoka, Eric R Olson, and Nirasha Ramchurren

## Supplementary Material

Additional file 1 **Table S1. Risk of Contralateral breast cancer associated with *FGFR2 *SNPs**. Table S2: Risk of Contralateral breast cancer associated with *FGFR2 *haplotypes. Click here for file
