# Supplementary material for: Single nucleotide polymorphisms associated with risk for contralateral breast cancer in the Women's Environment, Cancer, and Radiation Epidemiology (WECARE) Study
Source: Breast Cancer Res. 2011 Nov 17;13(6):R114. doi: 10.1186/bcr3057 (PMC3326556; doi:10.1186/bcr3057)
Supplement: Additional file 1 — Table S1. Risk of Contralateral breast cancer associated with FGFR2 SNPs. Table S2: Risk of Contralateral breast cancer associated with FGFR2 haplotypes. [file bcr3057-S1.DOC]

**Table S1. Risk of Contralateral breast cancer associated with *FGFR2* SNPs**

| ***FGFR2* SNP^a^** | **Risk**  **Allele** | **Allele**  **Freq.^b^** | **Per allele**  **Rate Ratio^c^**  **Trend (95% CI)** | **Heterozygous**  **Rate Ratio^c^**  **(95% CI)** | **Homozygous**  **Rate Ratio^c^**  **(95% CI)** |
| --- | --- | --- | --- | --- | --- |
|  |  |  |  |  |  |
| rs2981582 | A | .44 | 1.20 (1.04-1.40) | 1.26 (0.99-1.60) | 1.45 (1.08-1.95) |
| rs17102287 | C | .21 | 1.03 (0.86-1.23) | 1.00 (0.80-1.25) | 1.14 (0.69-1.88) |
| rs2981578 | C | .55^d^ | 1.28 (1.10-1.48) | 1.31 (1.00-1.72) | 1.64 (1.21-2.22) |
| rs1078806 | G | .46 | 1.21 (1.05-1.40) | 1.23 (0.97-1.56) | 1.47 (1.10-1.97) |
| rs17542768 | G | .10 | 0.82 (0.64-1.05) | 0.84 (0.64-1.09) | 0.58 (0.18-1.87) |
| rs3750817 | C | .61 | 1.21 (1.03-1.41) | 1.16 (0.84-1.61) | 1.43 (1.03-2.00) |

Abbreviations: CI=confidence interval

^a^ We used HapMap CEU data to select five haplotype tagging SNPs, in addition to the previously genotyped rs2981582 from a 25 kb region surrounding *FGFR2* intron 2 where the initial evidence of association was observed; these additional haplotype-tagging SNPs were genotyped in all 2102 available WECARE Study participants.

^b^Allele frequency in the WECARE Study population.

^c^Adjusted for exact age at diagnosis of the first primary and the weighting factor that accounts for the counter-matched design.

^d^For rs2981578, C is not the minor allele in this study population.

**Table S2: Risk of Contralateral breast cancer associated with *FGFR2* haplotypes.**

| ***FGFR2* haplotype^a^** | **Number**  **cases** | **Number**  **controls** | **Rate Ratio^b^**  **(95% CI)** |
| --- | --- | --- | --- |
|  |  |  |  |
| CAACCA | 9 | 13 | 0.8 (0.3-2.5) |
| CAGCCA | 237 | 489 | 1.0 (0.8-1.3) |
| CAACTA | 15 | 26 | 1.5 (0.7-3.6) |
| CAGCTA | 308 | 523 | 1.3 (1.1-1.6) |
| CAACTG | 92 | 191 | 1.0 (0.8-1.4) |
| CAATTG | 140 | 276 | 1.0 (0.7-1.2) |
| CAGCTG | 48 | 80 | 1.1 (0.7-1.7) |
| TAATTG | 273 | 584 | 0.8 (0.7-1.0) |
| TGATTG | 125 | 273 | 0.8 (0.6-1.1) |

Abbreviations: CI=confidence interval.

^a^Common haplotypes present in >1 % of the study population. Order of the SNPs: rs3750817, rs17542768, rs1078806, rs2981578, rs17102287, rs2981582.

^b^Adjusted for exact age at diagnosis of the first primary and the weighting factor that accounts for the counter-matched design; the referent group for each haplotype is comprised of those without that haplotype.
